# Sub-megabase resolution tiling (SMRT) array-based comparative genomic hybridization profiling reveals novel gains and losses of chromosomal regions in Hodgkin Lymphoma and Anaplastic Large Cell Lymphoma cell lines

**DOI:** 10.1186/1476-4598-7-2

**Published:** 2008-01-07

**Authors:** Faisal M Fadlelmola, Minglong Zhou, Ronald J de Leeuw, Nirpjit S Dosanjh, Karynn Harmer, David Huntsman, Wan L Lam, Diponkar Banerjee

**Affiliations:** 1 Centre for Translational and Applied Genomics (CTAG), Department of Pathology and Laboratory Medicine, British Columbia Cancer Agency, Vancouver Cancer Centre, Vancouver, BC, V5Z 4E6, Canada; 2 Department of Cancer Genetics and Developmental Biology, British Columbia Cancer Research Centre, Vancouver, BC, V5Z 1L3, Canada

## Abstract

**Background:**

Hodgkin lymphoma (HL) and Anaplastic Large Cell Lymphoma (ALCL), are forms of malignant lymphoma defined by unique morphologic, immunophenotypic, genotypic, and clinical characteristics, but both overexpress CD30. We used sub-megabase resolution tiling (SMRT) array-based comparative genomic hybridization to screen HL-derived cell lines (KMH2 and L428) and ALCL cell lines (DEL and SR-786) in order to identify disease-associated gene copy number gains and losses.

**Results:**

Significant copy number gains and losses were observed on several chromosomes in all four cell lines. Assessment of copy number alterations with 26,819 DNA segments identified an average of 20 genetic alterations. Of the recurrent minimally altered regions identified, 11 (55%) were within previously published regions of chromosomal alterations in HL and ALCL cell lines while 9 (45%) were novel alterations not previously reported. HL cell lines L428 and KMH2 shared gains in chromosome cytobands 2q23.1-q24.2, 7q32.2-q36.3, 9p21.3-p13.3, 12q13.13-q14.1, and losses in 13q12.13-q12.3, and 18q21.32-q23. ALCL cell lines SR-786 and DEL, showed gains in cytobands 5p15.32-p14.3, 20p12.3-q13.11, and 20q13.2-q13.32. Both pairs of HL and ALCL cell lines showed losses in 18q21.32-18q23.

**Conclusion:**

This study is considered to be the first one describing HL and ALCL cell line genomes at sub-megabase resolution. This high-resolution analysis allowed us to propose novel candidate target genes that could potentially contribute to the pathogenesis of HL and ALCL. FISH was used to confirm the amplification of all three isoforms of the trypsin gene (PRSS1/PRSS2/PRSS3) in KMH2 and L428 (HL) and DEL (ALCL) cell lines. These are novel findings that have not been previously reported in the lymphoma literature, and opens up an entirely new area of research that has not been previously associated with lymphoma biology. The findings raise interesting possibilities about the role of signaling pathways triggered by membrane associated serine proteases in HL and aggressive NHL, similar to those described in epithelial tumors.

## Background

Hodgkin lymphoma (HL) and Anaplastic Large Cell Lymphoma (ALCL), are forms of malignant lymphoma defined by unique morphologic, immunophenotypic, genotypic, and clinical characteristics, but both overexpress CD30 [1,2], a 120 kDa member of the TNF/NGFR family [3]. The aetiology and pathogenesis of these two disorders are incompletely understood. Hodgkin's lymphoma (HL), one of the most common malignant lymphomas in young adults, was first described by Thomas Hodgkin in 1832. The cellular composition of the tumor tissue in HL consists of a small number (approximately 0.1% to 1%) of neoplastic Hodgkin and Reed-Sternberg (HRS) cells surrounded by a sea of benign reactive cells of diverse lineage, with or without dense sclerosis. Although the molecular mechanisms that give rise to HRS cells remain to be fully elucidated, it is now established that such cells are derived from pre-apoptotic germinal centre B cells which have lost much of the B-lineage-specific gene expression [4,5].

Anaplastic large cell lymphoma (ALCL) is a type of non-Hodgkin lymphoma seen in both adults and children. Most cases are derived from T cells or "null" cells lacking T or B phenotypic or genotypic lineage markers. Up to 75% of ALCL cases harbour the translocation (2;5) (p23;q35) [6]. This translocation results in a fusion gene containing the nucleophosmin gene (NPM) and a receptor tyrosine kinase gene called anaplastic lymphoma kinase (ALK). The NPM-ALK fusion gene codes for a constitutively activated tyrosine kinase that behaves as an oncogene. Several variant translocations and fusion genes are now known. Anti-ALK antibodies can be used in formalin-fixed paraffin embedded tissue sections in order to identify ALCL cases with t(2;5) or variant translocations. Over 80% of paediatric ALCL and 30% of adult ALCL express ALK. ALK+ cases tend to be in younger patients with a better overall survival than ALK negative cases [7].

There are common morphological and/or phenotypic features between classic Hodgkin lymphoma (cHL) and some non-Hodgkin lymphoma (NHL), including primary mediastinal B cell lymphoma, diffuse large B cell lymphoma, and ALCL [8]. The term Hodgkin's-like anaplastic large cell lymphoma (HD-Like ALCL) was used in the Revised European American lymphoma (REAL) classification to describe borderline cases with features of both ALCL and classical HL (cHL) [9,10]. The World Health Organization (WHO) classification system does not recognise this category as an entity. For cases on the morphological borderline between HL and ALCL, the WHO classification system states that the expression of CD15 without the expression of T-cell antigens favors HL, whereas the absence of CD15 expression and the presence of T-cell antigens favors ALCL [11]. The exact pathobiologic differences between these two types of lymphoma, however, remain to be fully explained. To investigate these common features between HL and ALCL, we undertook a study of gene copy number alteration profiling of these lymphomas using array-based submegabase resolution comparative genomic hybridization in 2 HL and 2 ALCL cell lines.

It is possible to directly identify genes involved in chromosomal alterations in cell line model systems and then rapidly explore their significance as potential diagnostic and therapeutic targets and roles in human cancer progression. Comparative genomic hybridization (CGH) is a technique that permits the detection of chromosomal copy number changes without the need for cell culturing. Conventional CGH uses metaphase chromosomes and the method typically has a resolution of 10 Mb, suitable for simple loss or gain assessments [12]. In array-CGH well-defined arrayed sequences of DNA have replaced the metaphase chromosomes as the hybridization targets on glass slides [13]. This approach enables a quantitative measure of gene copy number alterations at high resolution and uses *in-silico *analysis to map them accurately and directly to chromosomal locations. Array-CGH is more sensitive than conventional CGH because genomic DNA arrays allow for the detection of several chromosomal abnormalities that are missed by conventional CGH [14-16]. Most recently, the whole human genome has been arrayed as 26,819 bacterial artificial chromosome (BAC) derived amplified fragment pools spotted in duplicate (53,638 elements) resulting in a Sub-Megabase Resolution Tiling (SMRT)-set re-array array with complete coverage of the sequenced human genome [17]. This method allows the detection of regions of loss or gain as small as 40–80 kb.

Previously published reports have used lower resolution methods to identify chromosomal alterations that may be involved in the pathogenesis of HL and/or ALCL. In the current study, SMRT-array-based-CGH was used to screen HL-derived cell lines (KMH2 and L428) and ALCL cell lines (DEL and SR-786) to identify, at high resolution, gene copy number alterations that may be involved in the pathogenesis of HL and ALCL. Gene copy number gains and losses were observed on several chromosomes in all four cell lines investigated in this study. These alterations not only confirmed previously published chromosomal regional aberrations in HL and ALCL, but also defined 9 novel abnormal regions. We identified several regions of amplification, many in loci not previously known to be amplified. In order to develop a more detailed molecular characterization of HL and ALCL cell lines, we used the Ontario Cancer Institute (OCI) Human 27 k cDNA microarrays to investigate genes differentially expressed in the same four cell models of HL and ALCL, in comparison to Universal Human Reference RNA (UHRR) [unpublished data]. The gene expression profiles were then examined for correlation with the gene copy number alterations identified by SMRT array-based CGH.

## Results

### Gene copy number profiles of the four HL and ALCL cell lines

**Figure 1:**
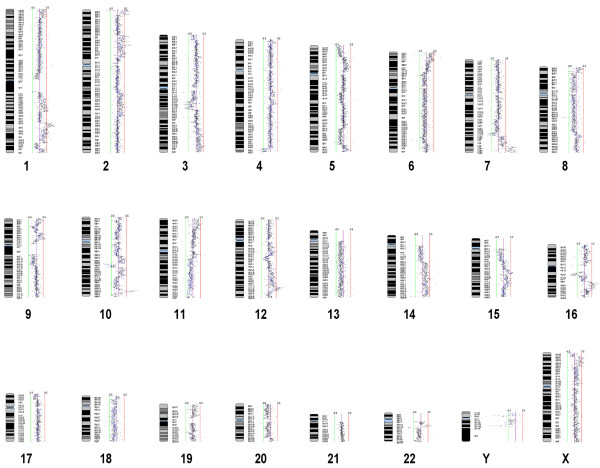
**Whole genome SMRT aCGH SeeGH karyogram of KMH2 cell line versus pooled normal male genomic DNA**. Each dot represents data from one BAC derived segment on the array. Data points to left and right of center purple line represent genetic losses and gains, respectively.

Gene copy number profiles of the four HL and ALCL cell lines were created by co-hybridizing differentially labeled sample DNA with reference male DNA on the a whole genome tiling resolution array that contains 26,819 BAC-derived amplified fragment pools spotted in duplicate. The analysis of 53,638 data points for each of the four cell lines facilitated the localization of altered chromosomal regions to within single BAC clones and the subsequent identification of genomic imbalances between regions. Gene copy number gains and losses were observed on at least 12 chromosomes in all four cell lines. Only those alterations recurring in at least two of the four cell lines were used to define minimally altered regions (MAR). A summary of genomic alterations recurring in HL and ALCL cell lines is shown in Table 1. These alterations defined 9 novel regions not previously reported in the literature. HL cell lines L428 and KMH2 shared gains in chromosome cytobands 2q23.1-q24.2, 7q32.2-q36.3, 9p21.3-p13.3, 12q13.13-q14.1, and losses in 13q12.13-q12.3, and 18q21.32-q23. ALCL cell lines SR-786 and DEL, showed gains in cytobands 5p15.32-p14.3, 20p12.3-q13.11, and 20q13.2-q13.32. Both pairs of HL and ALCL cell lines showed losses in 18q21.32-q23. Additional abnormalities were seen in individual cell lines, but were not common to both pairs of ALCL or HL cell lines. Figure 1 shows the whole genomic array CGH SeeGH karyogram of KMH2 cell line versus pooled normal male genomic DNA.

**Table 1:** Gains and Losses in HL ALCL cell lines

**Cytoband**	**KMH2**	**L428**	**DEL**	**SR-786**
1q25.2-q31.3	N	N	**G**	**G**
1q42.2-q43	L	N	**G**	**G**
2p16.2-p13.3	**G**	**G**	N	N
**2q23.1-q24.2**	**G**	**G**	N	N
2q24.2-q31.3	G	G	L	N
**5p15.32-p14.3**	L	N	**G**	**G**
5q14.1-q15	L	L	G	N
**7q11.1-q36.3**	**G**	**G**	**G**	N
8q23.3-24.12	**G**	**G**	N	N
9p24.3-p24.1	**G**	**G**	N	L
**9p21.3-p12**	**G**	**G**	N	N
11q22.1-q23.3	L	L	N	N
**12q13.13-q14.1**	**G**	**G**	N	N
**13q12.13-q12.3**	**L**	**L**	**G**	N
13q13.1-q21.32	L	L	N	N
13q31.1-q34	L	L	N	N
16q22.1-q24.1	**G**	**G**	N	N
**20p12.3-q13.11**	**L**	N	**G**	**G**
**20q13.2-q13.32**	**L**	N	**G**	**G**
**18q21.32-q23**	**L**	**L**	**L**	**L**

Novel regions are in underlined bold. L and G indicate losses and gains, respectively, in chromosomal regions in HL and ALCL cell lines, whereas N indicates no alteration was found.

### Correlation of gene copy number alterations and gene expression profiles

In order to study the overall impact of gene copy number alterations on gene expression, we analyzed the same four cell lines using the Ontario Cancer Institute (OCI) Human 27 k cDNA microarrays [unpublished data]. We evaluated mean gene expression and variability within array-based CGH altered regions and explored the correlation between the copy number alterations of each gene and its position within these regions as shown in Table 2. Only 35% (138/395) of expressed genes showed correlation with copy number alterations. Of these genes, 59% showed strong correlation whereas 41% showed weak correlation. These are similar to findings by other groups who found that the expression of genes in the same altered region correspond very differently to gain or loss of genetic material depending not only on a given gene's position but also on its regulation [18].

**Table 2:** Gains and Losses of the regions and the genes reported to be differentiallyexpressed.

**Cytoband**	**Size (Mb)**	**Minimal region boundary clones**	**Number of refseq genes in region**	**Reported Copy number alterations**	**Genes known/found to be differentially expressed**
1q25.2-q31.3	17.73	174994286–192721998	68	Gogusev et al. (2002)	**ANGPTL1, LHX4, PNF2, RGS1, NEK7, PCTRK3**, *CTSE*, *MYOG*, **RGS2**
1q42.2-q43	9.16	228621517–237777167	30	Gogusev et al. (2002)	**LGALS8, NID**, *MTR, OPN3, GNG4, TBCE*
2p16.2-p13.3	17.77	53587332–70354068	63	Joos et al. (2002)	*REL*, **PNPT1, RPS27A, XPO1**, *SLC1A4, MSH6, MGC15407*
**2q23.1-q24.2**	13.83	149314143–163139362	40	**current study**	**TNFAIP6, GCA, LY75, SLC25A12**, *DCL-1*, **ITGB6, RBMS1**
2q24.2-q31.3	18.76	163139362–181903843	81	Franke et al. (2001)	**MTX2, DPP4, ATP5G3, TLK1, STAT1**, *BBS5, SCRN3, NFE2L2*
**5p15.32-p14.3**	10	5257164–15261389	20	**current study**	**DNAH5, DDX4, MGC5309**, *ARFRP2, CTNND2, MYO10*
5q14.1-q15	18.99	79623141–98609560	48	Joos et al. (2003)	*C5ORF12, CACH-1, RPS23, ANKRD32, TBCA, KIAA0372, RHOBTB3*
**7q11.1-q36.3**	97.02	61369644–158387760	575	**current study**	**PRSS1, TRY6, PRSS2, CDK6, CLDN4, HSPB1, HGF, mTERF, ING3, TPST1**
8q23.3-24.12	5.08	116154379–121233686	19	Joos et al. (2003)	*MYC*, **RAD21, DEPDC6, THRAP6, DCC1**
9p24.3-p24.1	5.9	23994–5928516	28	Joos et al. (2003)	*JAK2*, **RANBP6, UHRF2, C9orf46, C9orf123**, *MLANA, RCL1, AK3L1*
**9p21.3-p12**	14.87	25192411–40063723	119	**current study**	**PRSS3, DNAJA1, RPS6, CCDC2**
11q22.1-q23.3	19.91	98410656–118317962	106	Falzetti et al. (1999)	*ATM*, *DDX6*, **CRYAB, PDGFD**
**12q13.13-q14.1**	8.64	53812697–62450135	114	**current study**	**DDIT3, DTX3, KRT6IRS, HOXC4, HOXC5, LEMD3, TBK1, USP15**
**13q12.13-q12.3**	5.49	25577047–31063440	25	**current study**	**GTF3A, MTIF3**, *PAN3, APRIN, BRCA2*
13q13.1-q21.32	35.16	31151386–66311960	108	Joos et al. (2002)	*Ufm1, C13orf1, RCBTB1, KBTBD6, NARG1L, DDX26, NUDT15*
13q31.1-q34	27.91	86156309–114062150	74	Joos et al. (2002)	*STK24, DCT, GPC5, DZI*, **EFNB2**, *TM9SF2, RASA3, LOC196541*
16q22.1-q24.1	19.68	65582104–85259588	170	Joos et al. (2002)	**CIRH1A, CKLFSF3, DNCL12, BMO39**
**20p12.3-q13.11**	33.93	8872139–42799402	185	**current study**	**PCNA, SNAP25, FKBP1A, SNPH, TH1L, SPAG4L, TDE1, SLC2A10, C20orf52, EYA2**
**20q13.2-q13.32**	6.95	50997916–57946927	31	**current study**	**TFAP2C**, *CDH4, ZFP64, GNAS*, **ATP5E**
**18q21.32-q23**	19.03	58644127–77673453	52	**current study**	***VPS4B, RTTN, PIGN*, ZCCHC2, BCL2, *FLJ25715, TXNL4A, ZNF516*, PARD6G**

Aligned with data from the University of California, Santa Cruz Human Browser April 2003 Freeze. Novel regions are in underlined bold. Genes in italic and underlined are those that previously been reported {Joos et al. 2003 [19]; Gogusev et al. [20]; Joos et al. 2002 [29]; Franke et al. [54]; Falzetti et al. [55]}, whereas those in bold and italic are those found to be over-expressed and down regulated, respectively, in the current study.

### The genomic alterations recurring in HL and ALCL

We first investigated whether the identified MARs included the chromosomal alterations previously reported in HL and ALCL cell lines. The previously reported regions that show chromosomal alterations are shown in Table 2. Our data confirm previous results from primary Hodgkin's tumors suggesting an important pathogenic role of *MYC *(8q24), *REL *(2p16) and *JAK2 *(9p24) in HL [19]. Even in the case of previously identified regions, the current study added further information because most of the previous reported cases looked into either HL or ALCL alone and also used lower resolution methods. The current study has advantages over previous studies because it used array-based CGH for both HL and ALCL cell lines and, in addition, used the same cell lines for gene expression in order to correlate those chromosomal copy number alterations with the gene expression profiling. For example, Gogusev et al. [20] detected amplicons on 1q21~q44 on the ALCL-derived DEL cell line as shown in Table 2. In our current study, we could resolve two specific regions; one on 1q25.2-q31.3 and the other on 1q42.2-q43 that showed copy number gains in ALCL cell lines (DEL and SR-786). 

The 1q25.2-q31.3 (17.73 Mb) gene-dense MAR of gain in ALCL encompasses 68 Refseq annotated genes. Among these, *LHX4 *(LIM homeobox 4) was found to be upregulated in the HL and ALCL cell lines. There is no published information regarding the role of *LHX4 *in lymphoma thus further analysis is required to determine the significance of increased expression of *LHX4 *in HL and ALCL. Recently, using quantitative real-time reverse-transcription PCR, it was found that the *LHX4 *mRNA is expressed at high levels in leukemic cells and in an acute lymphoblastic leukemia (ALL) cell line [21]. In addition to the *LHX4 *gene, other candidate genes located along the chromosomal region 1q25.2-q31.3, such as *PNF2*, *NEK7 *and *PCTRK3*, could also be involved in ALCL pathogenesis. The other previously reported region is the amplified MAR on 2q, spanning 18.76 Mb, containing 81 Refseq annotated genes. Our study identified *MTX2 *(Metaxin 2) as a highly expressed gene. MTX2 was found to be differentially overexpressed in HL cell lines compared to ALCL cell lines.

The 13q12.3-q12.3 (5.49 Mb) gene-dense MAR of gain in DEL cell line and loss in HL encompasses 25 Refseq annotated genes. Among these *GTF3A *(General transcription factor IIIA) was found to be upregulated in HL in comparison to ALCL cell lines. There is no published information regarding the role of *GTF3A *in lymphoma, therefore further analysis is required to determine the significance of *GTF3A *in HL and ALCL. A recent study identified *GTF3A *as down-regulated in Down syndrome leukocytes in comparison to normal controls [22], which might implicate *GTF3A *dysfunction in Down syndrome-associated acute myeloid leukemia.

The MAR 2q23.1-q24.2 (13.83 Mb) seems to be a region of gain in HL and it encompasses 40 Refseq annotated genes. Among these are *TNFAIP6*, also known as *TSG6 *(tumor necrosis factor, alpha-induced protein 6), *LY75 *(Lymphocyte antigen 75), *SLC25A12 *(Solute carrier family 25 (mitochondrial carrier, Aralar), member 12), and *GCA *(Grancalcin, EF-hand calcium binding protein). *TNFAIP6*, *LY75*, *SLC25A12 *and GCA were found to be differentially up-regulated in HL in comparison to ALCL cell lines. *TSG-6 *protein is known to form a complex with inter-alpha-inhibitor (IalphaI), a potent serine protease inhibitor, which may be immobilized via the hyaluronan (HA)-binding domain of TSG-6 protein in the HA-rich extracellular matrix of cartilage [23]. It is suggested that this mechanism might protect cartilage from extensive degradation even in the presence of acute inflammation. Further analysis is required to investigate the role of *TNFAIP6/TSG-6*, *LY75*, *SLC25A12*, and *GCA *in HL and ALCL pathogenesis. The 20p13.2-q13.32 (6.95 Mb) gene-dense MAR of gain in ALCL and loss in HL encompasses 31 Refseq annotated genes. Among these, *PCNA *(Proliferating cell nuclear antigen) and FKBP1A (FK506 binding protein 1A, 12 kDa) were found to be up-regulated in ALCL and HL cell lines.

The 7q11.1-q36.3 (97.02 Mb) gene-dense MAR of gain in HL and DEL cell lines encompasses 575 Refseq annotated genes. Among these *are CDK6 *(Cyclin-dependent kinase 6), *PRSS1 *(Protease, serine, 1 (trypsin 1)), *PRSS2 (*Protease, serine, 2 (trypsin 2)), *CLDN4 *(Claudin 4), *HSPB1 *(Heat shock 27 kDa protein 1), and *HGF *(Hepatocyte growth factor (hepapoietin A; scatter factor)). *PRSS1*, *PRSS2 *and *HSPB1 *were found to be overexpressed in HL cell lines, whereas *CLDN4 *was found to be overexpressed in both HL and ALCL cell lines. *CDK6 *and *HGF *were found to be overexpressed in the 2 HL cell lines and in the DEL (ALCL) cell line. Further study will obviously be essential to validate the *CDK6, HSPB1, PRSS1*, *PRSS2, HGF, and CLDN4 *expression at the protein level in HL and ALCL.

**Figure 2:**
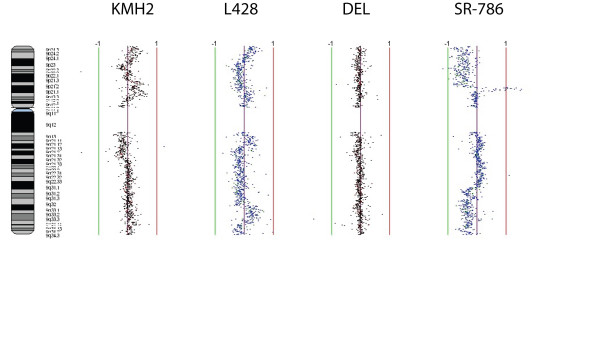
**Genomic profiles of chromosome 9 for the cell lines KMH2, L428, DEL and SR-786**. Data points to left and right of center purple line represents genetic losses and gains in the above mentioned cell lines. Green and red lines are scale bars at log2 ratios of -1.0 and 1.0 respectively.

**Figure 3:**
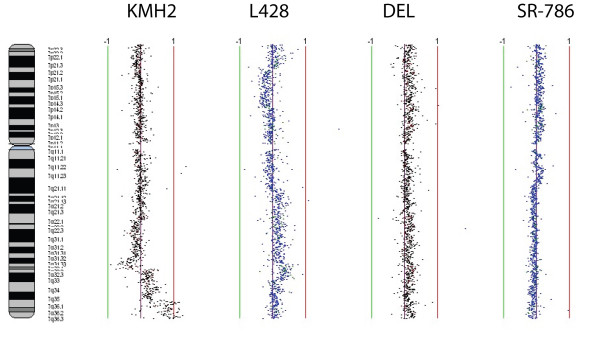
**Genomic profiles of chromosome 7 for the cell lines KMH2, L428, DEL and SR-786**. Data points to left and right of center purple line represents genetic losses and gains in the above mentioned cell lines. Green and red lines are scale bars at log2 ratios of -1.0 and 1.0 respectively.

**Figure 4:**
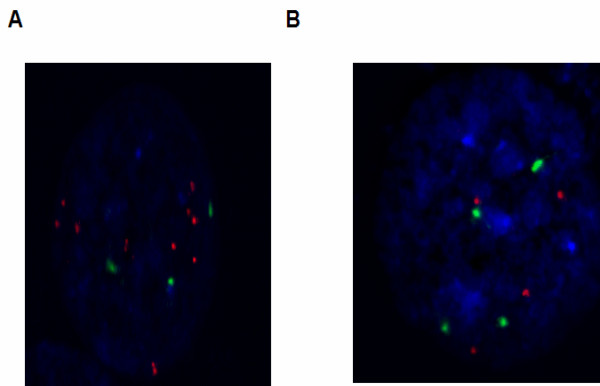
**Locus-specific FISH validation of genetic copy number alterations for Trypsin gene**. **(A) **Locus-specific FISH of 9p21.1-p12 amplification in KHM2 cell line. **(B) **Locus-specific FISH of 7q32.2-q36.3 non-amplification in SR-786 cell line. Orange and green signals indicate loci of spectrum orange and spectrum green labeled FISH probes, respectively.

Chromosomes 7 and 9 showed amplification of isoforms of the trypsin gene in the KMH2 (7q32.2-q36.3), L428 (7q34-q35) and DEL (7q11.1-q36.3) cell lines but not in SR-786 in the case of *PRSS1/PRSS2*, whereas *PRSS3 *showed amplification in HL cell lines (KMH2 at 9p21.1-p13.3 and L428 at 9p21.1-p12) but not in ALCL cell lines as shown in Figures 2 and 3. These findings were confirmed by FISH analysis as shown in Figure 4. These are novel findings that have not been previously reported in the lymphoma literature, and opens up an entirely new area of research that has not been associated with lymphoma biology. The observations raise interesting possibilities about the role of signaling pathways triggered by membrane associated serine proteases in HL and ALCL, similar to those implicated in epithelial tumors [24]. Confirmation of these findings could lead to novel therapeutic approaches in HL and NHL.

### Pathway mapping of the genes reported to be differentially expressed in HL and ALCL cell lines

**Figure 5:**
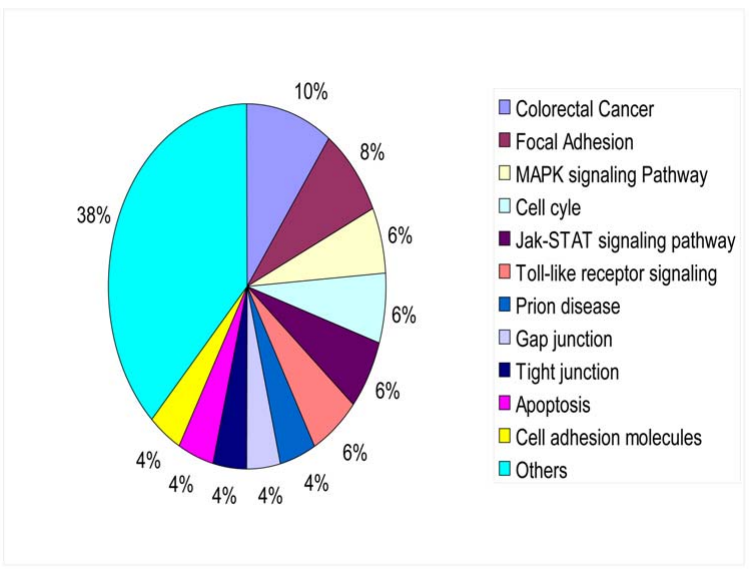
Pathways of the differentially expressed genes in HL-derived and ALCL-derived cell lines.

The list of the 137 genes we found to be differentially expressed in HL and ALCL cell lines (Table 2) were subjected to the Pathway-Express (PE) software [25]. PE generates a list of pathways that the submitted genes are involved in, complete with a p-value for each pathway, indicating the relative importance of each. After generating a list of pathways for the submitted list of genes from the Onto-Tools database, PE first calculates a perturbation factor PF (g) for each input gene. The PF takes into account the (i) normalized fold change of the gene and (ii) the number and amount of perturbation of genes downstream from it [26]. Fifty of these 137 deregulated genes in HL and ALCL were found to represent 12 main different pathways (Figure 5). Of these, pathways that included only 1 gene, were grouped together in the "others" category (38%). Genes involved in colorectal cancer were represented and corresponded to 10% of the total number of deregulated genes. Eight % of the genes are involved in encoding focal adhesion proteins and 6% of the genes are involved in the MAPK signaling pathway. We also found that 6% of the genes are involved in Jak/STAT signaling pathway, 6% in the cell cycle, 6% in Toll-like receptor signaling, 4% in encoding cell adhesion molecules and 4% in apoptosis.

### Functional profiling of the genes reported to be differentially expressed in HL and ALCL cell lines

The list of the 137 genes reported to be differentially expressed in HL and ALCL cell lines (Table 2) were also subjected to the Onto-Express (OE) software [27]. OE can be used to organize the list of differentially regulated genes into groups, allowing a better understanding of the underlying biological functions through the use of Gene Ontology (GO) database, complete with a p-value for each functional profile indicating the relative importance of each. A comprehensive list of these functional groups including a list of all genes can be found in [see Additional file 1].

## Discussion

Chromosomal aberrations can be studied using many different techniques, such as Comparative Genomic Hybridization (CGH), Fluorescence in Situ Hybridization (FISH), and Representational Difference Analysis (RDA). Although chromosome CGH quickly became a standard method for cytogenetic studies, technical limitations restrict its usefulness as a comprehensive screening tool. Recently, the resolution of Comparative Genomic Hybridizations has been greatly improved using microarray technology. Substitution of the chromosome targets by a matrix consisting of an ordered set of defined nucleic acid target sequences greatly enhances the resolution and simplifies the analysis procedure, both of which are prerequisites for a broad application of CGH as a research and diagnostic tool. Array CGH has provided significant contributions to our understanding of chromosomal changes associated with tumor development and progression, making it possible to detect 40–80 kb regions of chromosomal gain and loss covering the entire human genome in a single experiment [28].

The genetic alterations involved in the pathogenesis of HL are still largely unknown. HRS cells in most if not all cases show numerical chromosome aberrations, as well as amplifications and deletions of chromosomal sub-regions [29,30]. Several recurrent chromosomal alterations have been previously described but for most of these, identification of the relevant genes is still pending [29,31]. Recently, Kluiver et al. performed serial analysis of gene expression (SAGE) and array-based comparative genomic hybridization (aCGH) to identify genes involved in the pathogenesis of classical Hodgkin lymphoma (cHL) [32]. The comparison of SAGE libraries of cHL cell lines L428 and L1236 with those of germinal centre B cells revealed consistent overexpression of only 14 genes. In contrast, 141 genes were downregulated in both cHL cell lines, including many B cell and HLA genes and aCGH revealed gain of 2p, 7p, 9p, 11q and Xq and loss of 4q and 11q [32].

We studied tumor cell lines derived from HL and ALCL rather than primary lymphoma samples for the following reasons. Primary lymphomas are heterogeneous, with varying components such as infiltrating lymphocytes, invading blood vessels, and other stromal components which contribute to the extracted DNA and RNA and thus mask the signature of the neoplastic cells. The use of cell lines avoids the problem of having heterogeneous populations of cells complicating the analysis of the hybridization signals. Moreover, most of these cell lines preserve the phenotypic and differentiation related characteristics of lymphoma. Array-based-CGH was used to screen HL-derived cell lines (KMH2 and L428) and ALCL cell lines (DEL and SR-786) to identify chromosomal region gains and losses and gene copy number alterations that may reveal genes involved in the pathogenesis of HL and ALCL. Gene copy number gains and losses were observed on at least 12 chromosomes in all four cell lines investigated in this study. Assessment of copy number alterations with 26,819 DNA segments identified an average of 20 genetic alterations. These alterations defined 9 (45%) novel abnormal regions not previously reported in the literature. These novel regions may require further investigation in normal and tumor samples to eliminate the possibility of copy number variations within the normal human population. Of the recurrent MARs identified, 11 (55%) were identical to those determined in previously published studies of genetic alterations using lower resolution CGH methods than the sub-megabase resolution array-based CGH used in the current study.

The alterations in the main pathways responsible for controlling the cell cycle in HL have rarely been studied and poorly understood, mainly because the techniques used for molecular studies in this disease have been limited by the scarcity of malignant cells of interest [33]. The analysis of cell-cycle regulation in different types of lymphoid and epithelial neoplasms reveals a relationship whereby increased clinical aggressiveness is associated with the accumulation of genetic and epigenetic alterations [34]. In the current study, 3.6% of the genes reported to be differentially expressed in HL and ALCL cell lines were involved in cell cycle pathways. These include *CDK6*, *PCNA*, and *ATM*.

The mitogen-activated protein kinases (MAPKs) (also called extracellular signal-regulated kinase [ERK]) are a group of protein serine/threonine kinases that are activated in response to a variety of extracellular stimuli and mediate signal transduction from the cell surface to the nucleus [35,36]. Zheng et al. [37] reported that the active phosphorylated form of MAPK/ERK is aberrantly expressed in cultured and primary HD cells. In the current study, 3.6% of the genes were found to represent the MAPK signaling pathway. These are *DDIT3*, *HSPB1 *and *MYC*. Recently it was reported that Myxoid/round cell liposarcoma (MLS/RCLS) may develop from cell types other than preadipocytes because the fusion oncogene *FUS-DDIT3 *and the normal DDIT3 induce a liposarcoma phenotype when expressed in a primitive sarcoma cell line [38]. The MLS/RCLS oncogene *FUS-DDIT3 *is the result of a translocation derived gene fusion between the splicing factor *FUS *and *DDIT3*. *DDIT3 *and *FUS-DDIT3 *show opposing transcriptional regulation of IL8 and suggest that *FUS-DDIT3 *may affect the synergistic activation of promoters regulated by the CCAAT/enhancer binding protein (C/EBP) beta and NFkappaB [39].

Certain (2.4%) of the genes were found to be involved in encoding tight junction proteins. These include *CLDN4*, *PARD6G*. The claudin (CLDN) genes encode a family of proteins important in tight junction formation and function [40]. Recently, it was reported that *CLDN*, and *CLDN4 *are elevated in several epithelial malignancies such as those originating from the pancreas, bladder, thyroid, fallopian tubes, ovary, colon, breast, uterus, and prostate [40]. Surprisingly, our data showed overexpression of *CLDN4 *in HL and ALCL. The finding of expression of these claudins in other tumors warrant further investigation for *CLD4 *as well as other claudins in HL and ALCL pathogenesis.

Another 3.6% of the genes were determined to be involved in the Jak/STAT signaling pathway. These include *STAT1*, *JAK2 *and *MYC*. The STAT proteins (Signal Transducers and Activators of Transcription), were identified in the last decade as transcription factors which are critical in mediating virtually all cytokine driven signaling [41]. Several members of the STAT family of transcription factors, namely *STAT3*, *STAT5 *and *STAT6*, are frequently activated in HRS cells [42,43]. STATs can be activated by cytokine receptors via the *JAK *kinases, by receptor tyrosine kinases (RTKs) and by 7 transmembrane receptors [44]. In normal cells and in animals, ligand dependent activation of the STATs is a transient process, lasting for several minutes to several hours. In contrast, in many cancerous cell lines and tumors, where growth factor dysregulation is frequently at the heart of cellular transformation, the STAT proteins (in particular Stats 1, 3 and 5) are persistently tyrosine phosphorylated or activated [45]. STATs can be divided into two groups according to their specific functions [46]. One is made up of STAT2, STAT4, and STAT6, which are activated by a small number of cytokines and play a distinct role in the development of T-cells and in IFN signaling [46]. The other group includes STAT1, STAT3, and STAT5, activated in different tissues by means of a series of ligands and involved in IFN signaling, development of the mammary gland, response to GH, and embryogenesis [46]. This latter group of STATS plays an important role in controlling cell-cycle progression and apoptosis and thus contributes to oncogenesis. Although increased expression of STAT1 has been observed in many human neoplasia, this molecule can be considered a potential tumor suppressor, since it plays an important role in growth arrest and in promoting apoptosis [46]. On the other hand, STAT3 and 5 are considered to be oncogenes, since they bring about the activation of cyclin D1, c-Myc, and bcl-xl expression, and are involved in promoting cell-cycle progression, cellular transformation, and in preventing apoptosis. Recently, Cochet et al. reported that aberrant STAT activation in Hodgkin cells may promote cell survival and, as a consequence, facilitate oncogenic transformation [47].

Another interesting gene reported in this study is *ING3 *(inhibitor of growth family, member 3). The overexpression of *ING3 *was found to correlate with the gene copy number alterations at 7q11.1-q36.3 in HL cell lines (KMH2 and L428) and ALCL cell line (DEL) as shown in Table 1 and 2. The novel *ING *tumor-suppressor family proteins (*ING1-5*) have been recognized as the regulators of transcription, cell cycle checkpoints, DNA repair, apoptosis, cellular senescence, angiogenesis, and nuclear phosphoinositide signaling [48]. Using stable clones of melanoma cells overexpressing *ING3*, Wang et al. showed that overexpression of ING3 significantly promoted UV-induced apoptosis [48]. As there is no information reported in the literature about *ING3 *in HL and ALCL, further investigation is required to study the role of *ING3 *in HL and ALCL pathogenesis.

The use of the gene expression profiling of the same four cell lines used for array-based CGH proved to be useful in identifying differentially expressed genes in those chromosomal regions showing gene copy number alterations. A search for the involved genes located in these chromosomal regions can potentially shed light on the molecular pathogenesis of HL and ALCL. Our study found that different genes in the same altered region correspond very differently to gain and loss of genetic material, probably because of transcriptional regulation of each gene. A recent study compared gene expression levels with copy number alternations reported that gain of 2p and 9p did not result in overexpression of the proposed target genes *c-REL *and *JAK2*, but gain of two newly identified smallest region of overlap (SROs) on 7p and Xq did correlate with overexpression of *FSCN1 *and *IRAK1 *[32].

As can be seen from above (table 2), some of the genes in this study were previously reported to be involved in pathways of HL and ALCL pathogenesis. On the other hand, we propose that further analysis is required to investigate the role of overexpression of the other deregulated genes we have identified, that were not previously reported to be dysregulated in HL and ALCL.

## Conclusion

We used sub-megabase resolution tiling array-based comparative genomic hybridization to screen HL-derived cell lines (KMH2 and L428) and ALCL cell lines (DEL and SR-786) to identify disease-associated gene copy number gains and losses. We identified 9 novel alterations in regions not previously reported. This study is considered to be the first one in describing HL and ALCL cell line genomes at sub-megabase resolution. This high-resolution analysis allowed us to propose novel candidate genes that could potentially contribute to the pathogenesis of HL and ALCL. FISH was used to confirm the amplification of all three isoforms of the trypsin gene (PRSS1/PRSS2/PRSS3) in HL and DEL cell lines. These are novel findings that have not been previously reported in the lymphoma literature, and opens up an entirely new field of research that has not been associated with lymphoma biology. Our findings raise interesting possibilities about the role of signaling pathways triggered by membrane associated serine proteases in the pathogenesis of HL and aggressive NHL, similar to those implicated in epithelial tumors. Future validation studies on the copy number changes of the relevant genes in the 9 novel regions, gene expression and the protein expression levels in primary lymphoma tumor tissue are needed to verify our results and to identify new mechanisms in the pathogenesis of lymphomas and as potential new diagnostic and prognostic biomarkers.

## Methods

### Cell lines, Cell Culture conditions and DNA extraction

The human HL-derived cell lines KM-H2, L-428 and the ALCL cell lines DEL and SR-786 were obtained from the Deutsche ammlung von Mikrooganism und Zellkulturen GmbH (Braunschweig, Germany). All cell lines were cultured in RPMI 1640 supplemented with 10% heat-inactivated fetal bovine serum (Cansera Inc., Etiobicoke, ON, Canada), L-glutamine and penicillin and streptomycin in a humid environment of 5% CO2 at 37°C to a density of 10^6 ^cells/ml. Genomic DNA was extracted via standard proteinase K/RNAse treatment and phenol-chloroform extraction.

### Array CGH

Tiling path array comparative genomic hybridization was performed with a slight modification to the method described previously [49-51]. Briefly, 200 ng of test and reference DNA were separately labeled with Cyanine3-dCTP and Cyanine5-dCTP (Perkin Elmer Life Sciences Inc., Boston, MA, USA), respectively, via random priming at 37°C for 16–18 h in the dark. Labeled sample and reference DNA probes were combined, precipitated, re-suspended, blocked and hybridized onto BAC arrays containing 26,819 clones spotted in duplicate on a single slide. After hybridization, arrays were washed, rinsed, and dried using an oil free air stream. Slides were scanned using a charge-coupled device (CCD) based imaging system (arrayWoRx eAuto, Applied Precision, Issaquah, WA, USA). Images were analyzed using SoftWoRx Tracker Spot Analysis software (Applied Precision). The ratios were normalized using a stepwise normalization technique [52] to remove any biases added from non-biological sources.

Custom software (SeeGH) was used to visualize normalized data as log_2 _ratio plots. Genomic alterations were classified as homozygous deletions, loss, normal, gain, and amplification for ratios < -1.0, -1.0 to -0.15, -0.15 to 0.15, 0.15 to 1.0, and >1.0 respectively as previously described [50].

### Fluorescence in situ hybridization (FISH)

Bacterial artificial chromosome (BAC) clones N0520H11 and N0075N03 for PRSS1/2 and PRSS3, respectively, were obtained from Dr. Wan L. Lam at the BC Cancer Research Centre, Vancouver, BC, Canada. BAC DNA isolation was carried out using the standard laboratory method of rapid alkaline lysis maxipreparation. The chromosome 7 and 9 a-satellite plasmids and BAC DNA were labeled directly with SpectrumGreen-dUTP^® ^and SpectrumOrange-dUTP^® ^(Vysis, Downers Grove, IL, USA), respectively, using the Vysis nick translation kit (Vysis) in accordance with the manufacturer's instructions. Slide preparations of the cell lines were fixed using methanol:acetic acid (3:1), and dual-color FISH was performed. The labeled slides were counterstained with 4', 6-diamidino-2-phenylindole (DAPI; Sigma), mounted with antifade (Vysis), and stored at -20°C. At least 100 nuclei were evaluated in each sample. For all slides, FISH signals and patterns were detected using a Zeiss Axioplan Fluorescence microscope. Signals were scored manually and images were captured using Metasystems Isis FISH imaging software (MetaSystems Group, Inc. Belmont, MA). The analysis of trypsin gene amplification was conducted by counting the number of probe signals within 100 cells and calculating the average probe signals per cell. A ratio ≥ 2.0 of the probe signal to chromosome 7 in case of PRSS1/PRSS2 and chromosome 9 in case of PRSS3 was considered as positive for amplification using the criteria reported previously [53].

## Competing interests

The author(s) declare that they have no competing interests.

## Authors' contributions

All authors have read and approved the final version of the manuscript.

FF participated in the design of the study, participated in the array-CGH experiments, carried out the FISH analysis, interpreted the results, and prepared the manuscript. MZ contributed to the scientific discussion and manuscript preparation. RL carried out the array-CGH analysis, interpreted the results, and contributed to manuscript preparation. ND participated in array-CGH data analysis. KH participated in the FISH studies. DH contributed to FISH analysis, scientific discussion and manuscript preparation. WL participated in the design of the study, provided the array-CGH, and contributed in the preparation of the manuscript. DB participated in the design of the study and was responsible for its coordination, interpreted the results independently of the author 1, and contributed in the preparation of the manuscript.

## Supplementary Material

Additional file 1 A comprehensive list of the functional profiling of the genes. The data provided represent the functional profiling of the genes reported to be differentially expressed in HL and ALCL cell lines. Click here for file
