# Supplementary material for: Sub-megabase resolution tiling (SMRT) array-based comparative genomic hybridization profiling reveals novel gains and losses of chromosomal regions in Hodgkin Lymphoma and Anaplastic Large Cell Lymphoma cell lines
Source: Mol Cancer. 2008 Jan 7;7:2. doi: 10.1186/1476-4598-7-2 (PMC2254646; doi:10.1186/1476-4598-7-2)
Supplement: Additional file 1 — A comprehensive list of the functional profiling of the genes. The data provided represent the functional profiling of the genes reported to be differentially expressed in HL and ALCL cell lines. [file 1476-4598-7-2-S1.pdf]

Additional file 1: Functional profiling of the genes reported to be differentially expressed in HL and ALCL cell lines

Gene ID	Gene Name
**Zinc ion binding (10)**	
ZCCHC2	zinc finger, CCHC domain containing 2
ZNF516	zinc finger protein 516
DNAJA1	DnaJ (Hsp40) homolog, subfamily A, member 1
UHRF2	ubiquitin-like, containing PHD and RING finger domains, 2
DTX3	deltex 3 homolog (Drosophila)
RASA3	RAS p21 protein activator 3
ZFP64	zinc finger protein 64 homolog (mouse)
DCT	dopachrome tautomerase (dopachrome delta-isomerase, tyrosine-related
GTF3A	General transcription factor IIIA
ING3	inhibitor of growth family, member 3
**Transferase activity (13)**	
AK3L1	adenylate kinase 3-like 1
TPST1	tyrosylprotein sulfotransferase 1
MTR	5-methyltetrahydrofolate-homocysteine methyltransferase
TBK1	TANK-binding kinase 1
JAK2	Janus kinase 2 (a protein tyrosine kinase)
ATM	ataxia telangiectasia mutated (includes complementation groups A, C
STK24	serine/threonine kinase 24 (STE20 homolog, yeast)
NEK7	NIMA (never in mitosis gene a)-related kinase 7
CDK6	cyclin-dependent kinase 6
PNPT1	polyribonucleotide nucleotidyltransferase 1
TLK1	tousled-like kinase 1
LOC196541	Hypothetical protein LOC196541
PAN3	PAN3 polyA specific ribonuclease subunit homolog (S. cerevisiae)
**Transcription factor activity (9)**	
NFE2L2	nuclear factor (erythroid-derived 2)-like 2
DDIT3	DNA-damage-inducible transcript 3
MYOG	myogenin (myogenic factor 4)
STAT1	signal transducer and activator of transcription 1, 91kDa
MYC	v-myc myelocytomatosis viral oncogene homolog (avian)
REL	v-rel reticuloendotheliosis viral oncogene homolog (avian)
TFAP2C	transcription factor AP-2 gamma (activating enhancer binding protein 2 gamma)
HOXC5	homeobox C5
HOXC4	homeobox C4

Additional file 1 (continued)

Gene ID	Gene Name
**Signal transducer activity (7)**	
STAT1	signal transducer and activator of transcription 1, 91kDa
GNAS	GNAS complex locus
REL	v-rel reticuloendotheliosis viral oncogene homolog (avian)
TBK1	TANK-binding kinase 1
GNG4	guanine nucleotide binding protein (G protein), gamma 4
RGS1	regulator of G-protein signalling 1
RGS2	regulator of G-protein signalling 2, 24kDa
**Protein serine/threonine kinase activity (6)**	
ATM	ataxia telangiectasia mutated (includes complementation groups A, C
CDK6	cyclin-dependent kinase 6
NEK7	NIMA (never in mitosis gene a)-related kinase 7
TLK1	tousled-like kinase 1
TBK1	TANK-binding kinase 1
STK24	serine/threonine kinase 24 (STE20 homolog, yeast)
**Protein binding (27)**	
PARD6G	par-6 partitioning defective 6 homolog gamma (C. elegans)
CTNND2	catenin (cadherin-associated protein), delta 2 (neural
UHRF2	ubiquitin-like, containing PHD and RING finger domains, 2
XPO1	exportin 1 (CRM1 homolog, yeast)
NARG1L	NMDA receptor regulated 1-like
RHOBTB3	Rho-related BTB domain containing 3
DTX3	deltex 3 homolog (Drosophila)
ING3	inhibitor of growth family, member 3
SNAP25	synaptosomal-associated protein, 25kDa
RCBTB1	regulator of chromosome condensation (RCC1) and BTB (POZ) domain
PCNA	proliferating cell nuclear antigen
CDK6	cyclin-dependent kinase 6
TNFAIP6	tumor necrosis factor, alpha-induced protein 6
TH1L	TH1-like (Drosophila)
VPS4B	vacuolar protein sorting 4 homolog B (S. cerevisiae)
ITGB6	integrin, beta 6
KBTBD6	kelch repeat and BTB (POZ) domain containing 6
HSPB1	heat shock 27kDa protein 1
FKBP1A	FK506 binding protein 1A, 12kDa
BRCA2	breast cancer 2, early onset
CDH4	cadherin 4, type 1, R-cadherin (retinal)
RAD21	RAD21 homolog (S. pombe)
PRSS3	protease, serine, 3 (mesotrypsin)
STAT1	signal transducer and activator of transcription 1, 91kDa
MYC	v-myc myelocytomatosis viral oncogene homolog (avian)
ATM	ataxia telangiectasia mutated (includes complementation groups A, C
ATP5E	ATP synthase, H+ transporting, mitochondrial F1 complex, epsilon

Additional file 1 (continued)

Gene ID	Gene Name
**Nucleotide binding (17)**	
AK3L1	adenylate kinase 3-like 1
DNAH5	dynein, axonemal, heavy polypeptide 5
GNAS	GNAS complex locus
LEMD3	LEM domain containing 3
TBK1	TANK-binding kinase 1
JAK2	Janus kinase 2 (a protein tyrosine kinase)
MSH6	mutS homolog 6 (E. coli)
STK24	serine/threonine kinase 24 (STE20 homolog, yeast)
NEK7	NIMA (never in mitosis gene a)-related kinase 7
CDK6	cyclin-dependent kinase 6
TLK1	tousled-like kinase 1
MYO10	myosin X
DDX4	DEAD (Asp-Glu-Ala-Asp) box polypeptide 4
DDX6	DEAD (Asp-Glu-Ala-Asp) box polypeptide 6
RBMS1	RNA binding motif, single stranded interacting protein 1
VPS4B	Vacuolar protein sorting 4 homolog B (S. cerevisiae)
PAN3	PAN3 polyA specific ribonuclease subunit homolog (S. cerevisiae)
**Metal ion binding (12)**	
ZCCHC2	zinc finger, CCHC domain containing 2
ZNF516	zinc finger protein 516
MTR	5-methyltetrahydrofolate-homocysteine methyltransferase
DNAJA1	DnaJ (Hsp40) homolog, subfamily A, member 1
UHRF2	ubiquitin-like, containing PHD and RING finger domains, 2
ATP5E	ATP synthase, H+ transporting, mitochondrial F1 complex, epsilon
DTX3	deltex 3 homolog (Drosophila)
RASA3	RAS p21 protein activator 3
ZFP64	zinc finger protein 64 homolog (mouse)
DCT	dopachrome tautomerase (dopachrome delta-isomerase, tyrosine-related
GTF3A	General transcription factor IIIA
ING3	inhibitor of growth family, member 3
**Hydrolase activity (8)**	
DDX4	DEAD (Asp-Glu-Ala-Asp) box polypeptide 4
ATP5E	ATP synthase, H+ transporting, mitochondrial F1 complex, epsilon
NUDT15	nudix (nucleoside diphosphate linked moiety X)-type motif 15
DDX6	DEAD (Asp-Glu-Ala-Asp) box polypeptide 6
EYA2	eyes absent homolog 2 (Drosophila)
PIGN	phosphatidylinositol glycan, class N
PRSS1	protease, serine, 1 (trypsin 1)
ACOT12	Acyl-CoA thioesterase 12
**DNA binding (8)**	
ATM	ataxia telangiectasia mutated (includes complementation groups A, C
RBMS1	RNA binding motif, single stranded interacting protein 1
ZFP64	zinc finger protein 64 homolog (mouse)

Additional file 1 (continued)

Gene ID	Gene Name
PCNA	proliferating cell nuclear antigen
UHRF2	ubiquitin-like, containing PHD and RING finger domains, 2
MTERF	mitochondrial transcription termination factor
GTF3A	General transcription factor IIIA
ZNF516	zinc finger protein 516
**Calcium ion binding (8)**	
STAT1	signal transducer and activator of transcription 1, 91kDa
GCA	grancalcin, EF-hand calcium binding protein
PRSS3	protease, serine, 3 (mesotrypsin)
SLC25A12	solute carrier family 25 (mitochondrial carrier, Aralar), member 12
HGF	hepatocyte growth factor (hepapoietin A; scatter factor)
PRSS2	protease, serine, 2 (trypsin 2)
CDH4	cadherin 4, type 1, R-cadherin (retinal)
PRSS1	protease, serine, 1 (trypsin 1)
**ATP binding (15)**	
AK3L1	adenylate kinase 3-like 1
DNAH5	dynein, axonemal, heavy polypeptide 5
TBK1	TANK-binding kinase 1
JAK2	Janus kinase 2 (a protein tyrosine kinase)
MSH6	mutS homolog 6 (E. coli)
STK24	serine/threonine kinase 24 (STE20 homolog, yeast)
NEK7	NIMA (never in mitosis gene a)-related kinase 7
CDK6	cyclin-dependent kinase 6
TLK1	tousled-like kinase 1
MYO10	myosin X
DDX4	DEAD (Asp-Glu-Ala-Asp) box polypeptide 4
VPS4B	vacuolar protein sorting 4 homolog B (S. cerevisiae)
PAN3	PAN3 polyA specific ribonuclease subunit homolog (S. cerevisiae)
DDX6	DEAD (Asp-Glu-Ala-Asp) box polypeptide 6
ACOT12	Acyl-CoA thioesterase 12
**Binding (5)**	
RTTN	rotatin
KIAA0372	Hypothetical protein KIAA0372
RANBP6	RAN binding protein 6
SLC25A12	solute carrier family 25 (mitochondrial carrier, Aralar), member 12
APRIN	androgen-induced proliferation inhibitor
**Nucleic acid binding (5)**	
ZCCHC2	zinc finger, CCHC domain containing 2
DDX4	DEAD (Asp-Glu-Ala-Asp) box polypeptide 4
ZFP64	zinc finger protein 64 homolog (mouse)
DDX6	DEAD (Asp-Glu-Ala-Asp) box polypeptide 6
BRCA2	breast cancer 2, early onset

Additional file 1 (continued)

Gene ID	Gene Name
**Peptidase activity (5)**	
USP15	ubiquitin specific peptidase 15
PRSS3	protease, serine, 3 (mesotrypsin)
CTSE	cathepsin E
PRSS2	protease, serine, 2 (trypsin 2)
PRSS1	protease, serine, 1 (trypsin 1)
**RNA binding (5)**	
RBMS1	RNA binding motif, single stranded interacting protein 1
DDX6	DEAD (Asp-Glu-Ala-Asp) box polypeptide 6
PNPT1	polyribonucleotide nucleotidyltransferase 1
XPO1	exportin 1 (CRM1 homolog, yeast)
GTF3A	General transcription factor IIIA
**Unfolding protein binding (4)**	
TBCE	tubulin-specific chaperone e
TBCA	tubulin-specific chaperone a
CRYAB	crystallin, alpha B
DNAJA1	DnaJ (Hsp40) homolog, subfamily A, member 1
**Receptor Activity (4)**	
ITGB6	integrin, beta 6
LY75	lymphocyte antigen 75
FKBP1A	FK506 binding protein 1A, 12kDa
OPN3	opsin 3 (encephalopsin, panopsin)
**Sequence-specific DNA binding (4)**	
DDIT3	DNA-damage-inducible transcript 3
HOXC5	homeobox C5
HOXC4	homeobox C4
NFE2L2	nuclear factor (erythroid-derived 2)-like 2
**Structural constituent of ribosome (4)**	
RPS27A	ribosomal protein S27a
KIAA0372	Hypothetical protein KIAA0372
RPS23	ribosomal protein S23
RPS6	ribosomal protein S6
**Transporter activity (4)**	
SLC2A10	solute carrier family 2 (facilitated glucose transporter), member 10
TM9SF2	transmembrane 9 superfamily member 2
SLC25A12	solute carrier family 25 (mitochondrial carrier, Aralar), member 12
ATP5G3	ATP synthase, H+ transporting, mitochondrial F0 complex, subunit C3

Additional file 1 (continued)

Gene ID	Gene Name
**Trypsin activity (3)**	
PRSS1	protease, serine, 1 (trypsin 1)
PRSS2	protease, serine, 2 (trypsin 2)
PRSS3	protease, serine, 3 (mesotrypsin)
**Protein-tyrosine kinase activity (3)**	
JAK2	Janus kinase 2 (a protein tyrosine kinase)
NEK7	NIMA (never in mitosis gene a)-related kinase 7
STK24	serine/threonine kinase 24 (STE20 homolog, yeast)
**Magnesium ion binding (3)**	
NEK7	NIMA (never in mitosis gene a)-related kinase 7
NUDT15	nudix (nucleoside diphosphate linked moiety X)-type motif 15
EYA2	eyes absent homolog 2 (Drosophila)
**GTPase activator activity (3)**	
RASA3	RAS p21 protein activator 3
RGS1	regulator of G-protein signalling 1
RGS2	regulator of G-protein signalling 2, 24kDa
**ATP-dependent helicase activity (3)**	
DDX4	DEAD (Asp-Glu-Ala-Asp) box polypeptide 4
DDX6	DEAD (Asp-Glu-Ala-Asp) box polypeptide 6
INTS6	integrator complex subunit 6
**Calmodulin binding (2)**	
RGS1	regulator of G-protein signalling 1
RGS2	regulator of G-protein signalling 2, 24kDa
**Chaperone binding (2)**	
TBCE	tubulin-specific chaperone e
TBCA	tubulin-specific chaperone a
**Double-stranded DNA binding (2)**	
RBMS1	RNA binding motif, single stranded interacting protein 1
MTERF	mitochondrial transcription termination factor
**GTP binding (3)**	
AK3L1	adenylate kinase 3-like 1
GNAS	GNAS complex locus
ARL15	ADP-ribosylation factor-like 15
**GTPase activity (2)**	
GNAS	GNAS complex locus
RHOBTB3	Rho-related BTB domain containing 3

Additional file 1 (continued)

Gene ID	Gene Name
**Hydrogen-transporting ATP/ase synthase activity, rotational mechanism (2)**	
ATP5E	ATP synthase, H+ transporting, mitochondrial F1 complex, epsilon
ATP5G3	ATP synthase, H+ transporting, mitochondrial F0 complex, subunit C3
**Identical protein binding (2)**	
BCL2	B-cell CLL/lymphoma 2
CLDN4	Claudin 4
**Isomerase activity (2)**	
DCT	dopachrome tautomerase (dopachrome delta-isomerase, tyrosine-related
FKBP1A	FK506 binding protein 1A, 12kDa
**Kinase activity (2)**	
AK3L1	adenylate kinase 3-like 1
STK24	serine/threonine kinase 24 (STE20 homolog, yeast)
**Protein dimerization activity (2)**	
DDIT3	DNA-damage-inducible transcript 3
NFE2L2	nuclear factor (erythroid-derived 2)-like 2
**Receptor binding (2)**	
JAK2	Janus kinase 2 (a protein tyrosine kinase)
ANGPTL1	angiopoietin-like 1
**Serine-type endopeptidase activity (2)**	
PRSS3	protease, serine, 3 (mesotrypsin)
HGF	hepatocyte growth factor (hepapoietin A; scatter factor)
**Single-stranded DNA binding (2)**	
RBMS1	RNA binding motif, single stranded interacting protein 1
BRCA2	breast cancer 2, early onset
**Structural molecule activity (2)**	
CTNND2	catenin (cadherin-associated protein), delta 2 (neural
CLDN4	Claudin 4
**Sugar binding (2)**	
LY75	lymphocyte antigen 75
LGALS8	lectin, galactoside-binding, soluble, 8 (galectin 8)
**3’-5’-exoribonuclease activity**	
PNPT1	polyribonucleotide nucleotidyltransferase 1
**Acetyltransferase activity**	
NARG1L	NMDA receptor regulated 1-like

Additional file 1 (continued)

Gene ID	Gene Name
**Actin binding**	
MYO10	myosin X
**Adenylate kinase activity**	
AK3L1	adenylate kinase 3-like 1
**Aminopeptidase activity**	
DPP4	dipeptidyl-peptidase 4 (CD26, adenosine deaminase complexing protein
**ATPase activity**	
DNAH5	dynein, axonemal, heavy polypeptide 5
**ATPase activity, coupled**	
VPS4B	vacuolar protein sorting 4 homolog B (S. cerevisiae)
**Catalytic activity**	
EYA2	eyes absent homolog 2 (Drosophila)
**Cathepsin E activity**	
CTSE	cathepsin E
**Cobalamin binding/ Cobalt ion binding**	
MTR	5-methyltetrahydrofolate-homocysteine methyltransferase
**Copper ion binding**	
DCT	dopachrome tautomerase (dopachrome delta-isomerase, tyrosine-related
**Cysteine-type endopeptidase activity**	
USP15	ubiquitin specific peptidase 15
**Damaged DNA binding**	
MSH6	mutS homolog 6 (E. coli)
**Dihydropteroate synthase activity**	
MTR	5-methyltetrahydrofolate-homocysteine methyltransferase
**Dipeptidyl-peptidase IV activity**	
DPP4	dipeptidyl-peptidase 4 (CD26, adenosine deaminase complexing protein
**DNA polymerase processivity factor activity**	
PCNA	proliferating cell nuclear antigen
**Dopachrome isomerase activity**	
DCT	dopachrome tautomerase (dopachrome delta-isomerase, tyrosine-related

Additional file 1 (continued)

Gene ID	Gene Name
**Ephrin receptor binding**	
EFNB2	ephrin-B2
**G-protein coupled photoreceptor activity**	
OPN3	opsin 3 (encephalopsin, panopsin)
**Growth factor activity**	
HGF	hepatocyte growth factor (hepapoietin A; scatter factor)
**Guanyl nucleotide binding**	
GNAS	GNAS complex locus
**Heat shock protein binding**	
DNAJA1	DnaJ (Hsp40) homolog, subfamily A, member 1
**Hematopoietin/interferon-class (D200 domain) cytokine receptor signal transducer activity**	
STAT1	signal transducer and activator of transcription 1, 91kDa
**Homocysteine S-methyltransferase activity**	
MTR	5-methyltetrahydrofolate-homocysteine methyltransferase
**Hyaluronic acid binding**	
TNFAIP6	tumor necrosis factor, alpha-induced protein 6
**Janus kinase activity**	
JAK2	Janus kinase 2 (a protein tyrosine kinase)
**Ligase activity**	
UHRF2	ubiquitin-like, containing PHD and RING finger domains, 2
**Lipid binding**	
ATP5G3	ATP synthase, H+ transporting, mitochondrial F0 complex, subunit C3
**Low-desnity lipoprotein receptor binding**	
DNAJA1	DnaJ (Hsp40) homolog, subfamily A, member 1
**Manganese ion binding**	
NUDT15	nudix (nucleoside diphosphate linked moiety X)-type motif 15
**Methionine synthase/** Methyltranaferase **activity**	
MTR	5-methyltetrahydrofolate-homocysteine methyltransferase
**Microtubule motor activity**	
DNAH5	dynein, axonemal, heavy polypeptide 5

Additional file 1 (continued)

Gene ID	Gene Name
**Motor activity**	
MYO10	myosin X
**Neutral amino acid transporter activity**	
SLC1A4	solute carrier family 1 (glutamate/neutral amino acid transporter), member 4
**Oxidoreductase activity**	
DCT	dopachrome tautomerase (dopachrome delta-isomerase, tyrosine-related
**Pepsin A activity**	
CTSE	cathepsin E
**Peptidyl-prolyl cis-trans isomerase activity**	
FKBP1A	FK506 binding protein 1A, 12kDa
**Polyribonucleotide nucleotidyltransferase activity**	
PNPT1	polyribonucleotide nucleotidyltransferase 1
**Prolyl oligopeptidase activity**	
DPP4	dipeptidyl-peptidase 4 (CD26, adenosine deaminase complexing protein
**Protein kinase activity**	
PAN3	PAN3 polyA specific ribonuclease subunit homolog (S. cerevisiae)
**Protein transporter activity**	
XPO1	exportin 1 (CRM1 homolog, yeast)
**Protein tyrosine phosphatase activity**	
EYA2	eyes absent homolog 2 (Drosophila)
**Protein tyrosine sulfotransferase activity**	
TPST1	tyrosylprotein sulfotransferase 1
**Rhodopsin-like receptor activity**	
OPN3	opsin 3 (encephalopsin, panopsin)
**Ribosome binding**	
NARG1L	NMDA receptor regulated 1-like
**RNA helicase activity**	
DDX6	DEAD (Asp-Glu-Ala-Asp) box polypeptide 6
**RNA polymerase III transcription factor activity**	
GTF3A	General transcription factor IIIA
**RNA splicing factor activity, transesterification mechanism**	
TXNL4A	thioredoxin-like 4A

Additional file 1 (continued)

Gene ID	Gene Name
**RNA-3'-phosphate cyclase activity**	
RCL1	RNA terminal phosphate cyclase-like 1
**S-adenosylmethionine-dependent methyltransferase activity**	
LOC196541	Hypothetical protein LOC196541
**Serine-type endopeptidase inhibitor activity**	
NFE2L2	nuclear factor (erythroid-derived 2)-like 2
**SH2 domain binding**	
JAK2	Janus kinase 2 (a protein tyrosine kinase)
**Table 3 (continued)**	
**Sodium:dicarboxylate symporter activity**	
SLC1A4	solute carrier family 1 (glutamate/neutral amino acid transporter), member 4
**Solute:cation symporter activity**	
SLC25A12	solute carrier family 25 (mitochondrial carrier, Aralar), member 12
**Structural constituent of eye lens**	
CRYAB	crystallin, alpha B
**Sugar porter activity**	
SLC2A10	solute carrier family 2 (facilitated glucose transporter), member 10
**Symporter activity**	
SLC1A4	solute carrier family 1 (glutamate/neutral amino acid transporter), member 4
**Syntaxin-1 binding**	
SNPH	syntaphilin
**Transcription corepressor activity**	
DDIT3	DNA-damage-inducible transcript 3
**Transcription termination factor activity**	
MTERF	mitochondrial transcription termination factor
**Translation initiation factor activity**	
MTIF3	mitochondrial translational initiation factor 3
**Transmembrance receptor activity**	
CLDN4	Claudin 4
**Ubiquitin thiolesterase activity**	
USP15	ubiquitin specific peptidase 15

Additional file 1 (continued)

Gene ID	Gene Name
**Cytokine activity**	
CMTM3	CKLF-like MARVEL transmembrane domain containing 3
**Serine esterase activity**	
ACOT12	Acyl-CoA thioesterase 12
**Transmembrane receptor activity**	
INTS6	integrator complex subunit 6
**Unknown function (33)**	
SPAG4L	sperm associated antigen 4-like
BBS5	Bardet-Biedl syndrome 5
UFM1	ubiquitin-fold modifier 1
SERINC3	serine incorporator 3
THRAP6	thyroid hormone receptor associated protein 6
PDGFD	platelet derived growth factor D
SCRN3	secernin 3
LHX4	LIM homeobox 4
TRY6	Trypsinogen C
DNCL12	DNCL12
CIRH1A	cirrhosis, autosomal recessive 1A (cirhin)
SERINC5	serine incorporator 5
DCC1	defective in sister chromatid cohesion homolog 1 (S. cerevisiae)
DCL-1	type I transmembrane C-type lectin receptor
KRT71	keratin 71
C20orf52	chromosome 20 open reading frame 52, nuclear gene encoding mitochondrial protein
DEPDC6	DEP domain containing 6
DZI	DZI
IFT74	intraflagellar transport 74 homolog (Chlamydomonas)
C9orf46	chromosome 9 open reading frame 46
GPC5	glypican 5
NID	NID
C9orf123	chromosome 9 open reading frame 123
PCTRK3	PCTRK3
PNF2	PNF2
FLJ25715	hypothetical protein FLJ25715
CCDC104	coiled-coil domain containing 104
MLANA	melan-A
C13orf1	chromosome 13 open reading frame 1
BMO39	BMO39
MTX2	metaxin 2
MED10	mediator of RNA polymerase II transcription, subunit 10 homolog (NUT2, S. cerevisiae)
ANKRD32	ankyrin repeat domain 32
